# The Combined Use of Phenothiazines and Statins Strongly Affects Doxorubicin-Resistance, Apoptosis, and Cox-2 Activity in Colon Cancer Cells

**DOI:** 10.3390/ijms20040955

**Published:** 2019-02-22

**Authors:** Kamila Środa-Pomianek, Krystyna Michalak, Anna Palko-Łabuz, Anna Uryga, Piotr Świątek, Michał Majkowski, Olga Wesołowska

**Affiliations:** 1 Department of Biophysics, Wroclaw Medical University, ul. Chalubinskiego 10, 50-368 Wroclaw, Poland; krystyna.michalak@umed.wroc.pl (K.M.); anna.palko-labuz@umed.wroc.pl (A.P.-Ł.); anna.uryga@umed.wroc.pl (A.U.); olga.wesolowska@umed.wroc.pl (O.W.); 2 Department of Chemistry of Drugs, Wroclaw Medical University, ul. Borowska 211, 50-556 Wroclaw, Poland; piotr.swiatek@umed.wroc.pl; 3 Confocal Microscopy Laboratory, Polish Center for Technology Development, ul. Stabłowicka 147, 54-066 Wrocław, Poland; michaljozefmajkowski@gmail.com

**Keywords:** phenothiazine derivatives, simvastatin, cancer multidrug resistance, apoptosis, cyclooxygenase-2 (COX-2), cancer combination therapy, ABCB1 (P-glycoprotein)

## Abstract

Since none of the multidrug resistance (MDR) modulators tested so far found their way into clinic, a novel approach to overcome the MDR of cancer cells has been proposed. The combined use of two MDR modulators of dissimilar mechanisms of action was suggested to benefit from the synergy between them. The effect of three phenothiazine derivatives that were used as single agents and in combination with simvastatin on cell growth, apoptosis induction, activity, and expression of cyclooxygenase-2 (COX-2) in doxorubicin-resistant colon cancer cells (LoVo/Dx) was investigated. Treatment of LoVo/Dx cells by phenothiazine derivatives combined with simvastatin resulted in an increase of doxorubicin cytotoxicity and its intracellular accumulation as compared to the treatment with phenothiazine derivatives that were used as single agents. Similarly, LoVo/Dx cells treated with two-component mixture of modulators showed the reduced expression of ABCB1 (P-glycoprotein) transporter and COX-2 enzyme, both on mRNA and protein level. Reduced expression of anti-apoptotic Bcl-2 protein and increased expression of pro-apoptotic Bax were also detected. Additionally, COX-2 activity was diminished, and caspase-3 activity was increased to a higher extent by phenothiazine derivative:simvastatin mixtures than by phenothiazine derivatives themselves. Therefore, the introduction of simvastatin strengthened the anti-MDR, anti-inflammatory, and pro-apoptotic properties of phenothiazines in LoVo/Dx cells.

## 1. Introduction

Despite the permanent progress in medical sciences, the effective treatment of many malignant diseases remains out of our reach. The resistance of tumor cells towards anticancer drugs, either intrinsic or developing during chemotherapy, is one of the biggest amongst the various obstacles in overcoming cancer. Drug-resistance of cancer cells may be a result of the overexpression of multispecific transporters of the ATP-binding cassette superfamily (ABC transporters), such as ABCB1 (P-glycoprotein) [1]. Moreover, resistant cells are often characterized by the altered response to molecular signals leading to apoptosis [2]. Since tumor development goes together with the inflammation process, it is accompanied by an increased expression of cyclooxygenase-2 (COX-2), which is the inducible form of cyclooxygenase that is usually not present in healthy tissue [3,4]. In addition, higher COX-2 levels have been observed in multidrug-resistant cells than in chemotherapy-sensitive cancer cells [5,6].

The idea to circumvent multidrug resistance (MDR) by the modulation of ABC transporters activity by low molecular weight modulators has emerged soon after the discovery of MDR phenomenon [7,8]. Despite the many efforts put in the development of effective MDR modulators, until now none of them found its way into clinic. This was mainly due to the impossibility to reach efficient concentration of the modulators in the plasma, intolerable side effects, as well as the interaction of MDR modulators with various off-targets (e.g., cytochromes) [9]. In the present work, we propose a novel approach to overcome drug resistance of cancer cells. Instead of using a single MDR modulator, we suggest the combined use of two compounds of a dissimilar mechanism of action to benefit from the synergy between them. An additional advantage of our approach would be the possible reduction of the concentrations of the modulators that are required to obtain the desired effect and thus to minimize the risk of side effects. Here, we demonstrate the strong reduction of doxorubicin (Dox) resistance of human adenocarcinoma cells (LoVo/Dx) by the combination of phenothiazine derivatives with simvastatin.

Antipsychotic properties of phenothiazine derivatives were discovered more than 60 years ago and their introduction to the clinic has revolutionized psychiatry [10]. Phenothiazine derivatives were among the first group of MDR modulators that have been recognized [11]. The inhibition of the transport activity of ABCB1 protein was suggested to be responsible for their anti-MDR properties [12,13]. Such compounds as trifluoperazine, fluphenazine, and thioridazine turned out to be efficient MDR reversing agents when used in vitro [14,15], however no phenothiazine derivative proved to be useful in the in vivo settings [16].

Statins are specific inhibitors of the major enzyme of cholesterol biosynthesis pathway, 3-hydroxy-3-methyl-glutaryl-coenzyme A reductase [17]. They belong to the most popular drugs that are prescribed to decrease the concentration of cholesterol in plasma and to reduce the risk of the development of cardiovascular diseases. Diverse anticancer properties of statins have been reported, including their ability to influence the expression of proteins that are engaged in the regulation of cell cycle and their ability to induce apoptosis in various cancer cell lines [18,19,20,21].

In the present work, we demonstrate that the treatment of Dox-resistant human colon adenocarcinoma cells LoVo/Dx by phenothiazine derivatives combined with simvastatin results in the increase of Dox-induced cytotoxicity as well as its intracellular accumulation as compared to the treatment with phenothiazine derivatives alone. Similarly, LoVo/Dx cells that were treated with two-component mixture of modulators showed the reduced expression of ABCB1 transporter, COX-2 enzyme, and anti-apoptotic Bcl-2 protein, whereas the expression of pro-apoptotic Bax protein was increased. Additionally, COX-2 activity was diminished and caspase-3 activity raised to a higher extent due to treatment with phenothiazine derivative:simvastatin mixtures than with phenothiazine derivatives alone. It was concluded that the introduction of simvastatin to LoVo/Dx cells that were treated by phenothiazine-type modulators strengthened their anti-MDR, anti-inflammatory, and pro-apoptotic properties.

## 2. Results

### 2.1. Doxorubicin Cytotoxicity

**Figure 1:**
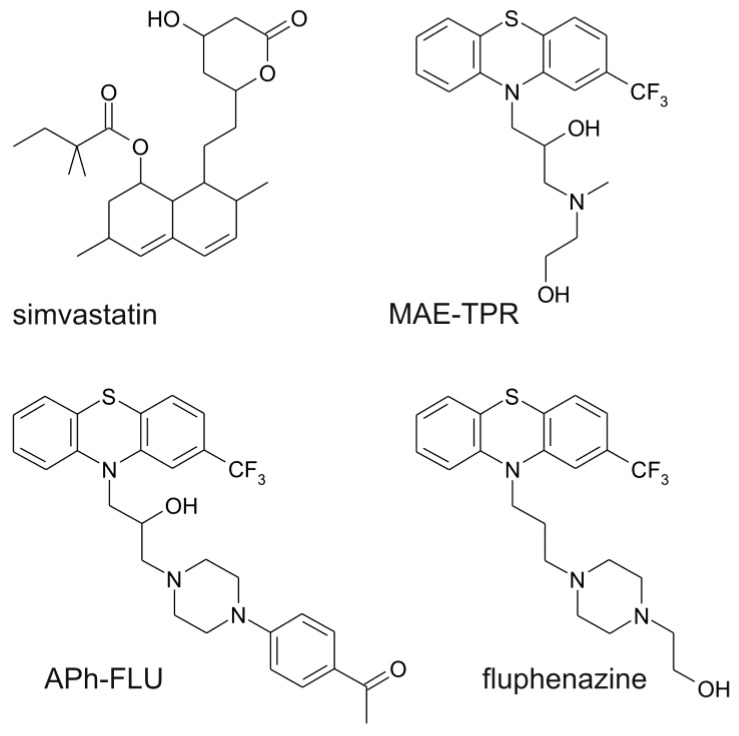
Chemical structures of the studied modulators.

**Figure 2:**
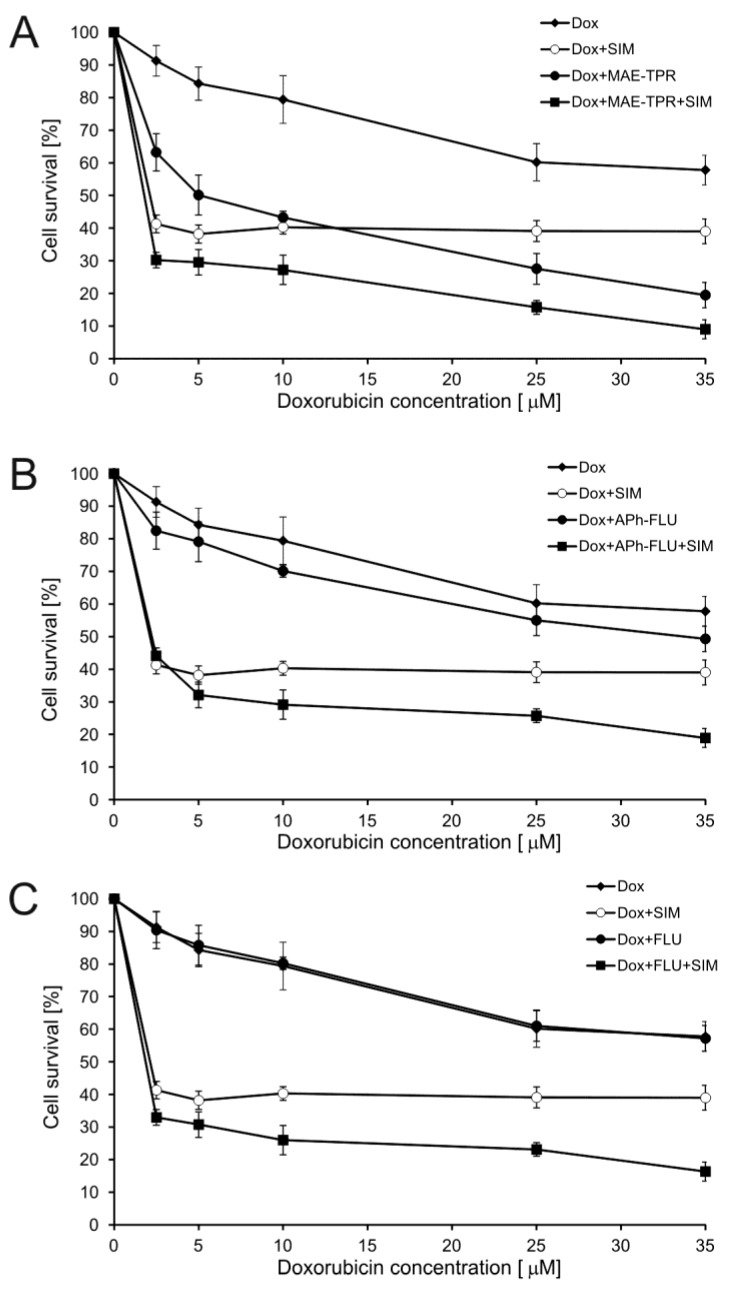
Cytotoxicity of doxorubicin (Dox) (diamonds), Dox, combined with simvastatin (SIM) at 2.5 μM (empty circles), Dox combined with phenothiazine derivative at 2.5 μM (full circles) and Dox combined with phenothiazine derivative at 2.5 μM and simvastatin at 2.5 μM (squares) to LoVo/Dx cells. The means of three experiments ± SD are presented.

The model that was used in the present work was doxorubicin-resistant subline LoVo/Dx (IC_50 Dox_ = 30.0 ± 3.7 μM) derived from human adenocarcinoma cell line LoVo (IC_50 Dox_ = 4.0 ± 0.9 μM). Simvastatin applied both at the concentration of 2.5 μM [22] and 10-[3-(*N*-2-hydroxyethyl-*N*-methylamino)-2-hydroxypropyl]-2-trifluoromethylphenothiazine (MAE-TPR) at 5 μM [23] was previously demonstrated to increase the cytotoxic effect of Dox on LoVo/Dx cells (for structures of the modulators used, see Figure 1). Here, the influence of simvastatin, fluphenazine (FLU), and its two derivatives MAE-TPR and 10-{3-[4-(4-acetylphenyl)piperazin-1-yl]-2-hydroxypropyl}-2-trifluoromethylphenothiazine dihydrochloride (APh-FLU) on the cytotoxicity of Dox on LoVo/Dx cells was tested. Each of the modulators was first applied alone, and then phenothiazine derivatives were applied in combination with simvastatin (Figure 2). All of the modulators were used at the concentration of 2.5 μM, at which their cytotoxicity to LoVo/Dx cells was previously shown to be less than 10% [22,24]. Simvastatin and MAE-TPR used alone significantly increased Dox cytotoxicity (Figure 2A). On the other hand, the influence of APh-FLU and FLU on the growth of Dox-treated LoVo/Dx cells was negligible (Figure 2B,C, respectively). In the case of all phenothiazine derivatives, the addition of simvastatin resulted in significant inhibition of cellular growth as compared to the treatment with Dox:phenothiazine and Dox:simvastatin combinations. When dose and effect data that were obtained from SRB assay were subjected to CompuSyn analysis, the synergy was observed between Dox and simvastatin, as well between Dox and MAE-TPR (Table 1). Simultaneous application of Dox, simvastatin and any of the phenothiazine derivatives produced CI values that were lower than in the case of two-component mixtures.

The difference in cytotoxicity between Dox and Dox, combined with the modulator, was statistically significant (*p* < 0.05, as determined by Student’s *t*-test) for simvastatin and MAE-TPR in the whole concentration range, but not for APh-FLU and FLU. For all phenothiazine derivatives, the addition of simvastatin resulted in statistically significant (*p* < 0.05, as determined by Student’s *t*-test) inhibition of cellular growth as compared to the treatment with Dox:phenothiazines and Dox:SIM combinations.

### 2.2. Doxorubicin Intracellular Accumulation

**Figure 3:**
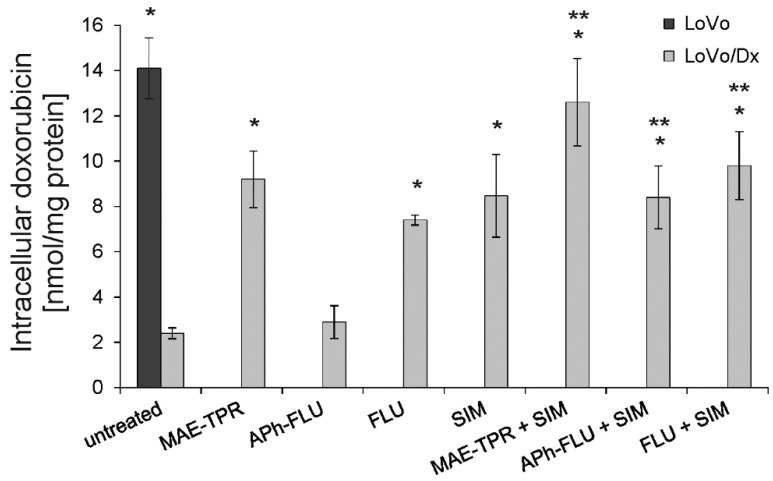
Intracellular doxorubicin (Dox)accumulation in LoVo (dark grey bars) and Lovo/DX cells (light grey bars) treated with phenothiazine derivatives, simvastatin (SIM) and phenothiazine derivatives in combination with simvastatin. Means of three experiments ± SD are presented. The statistically significant differences from the untreated LoVo/Dx control were determined using Student’s *t*-test (* *p* < 0.05). The statistically significant differences between the samples containing phenothiazine derivative as a single agent and samples with phenothiazine derivative combined with simvastatin were also determined using the Student’s *t*-test (** *p* < 0.05).

The influence of phenothiazine derivatives on human adenocarcinoma cells was further studied by analyzing the accumulation of Dox within the cells. Intracellular accumulation of the drug was significantly higher in the untreated LoVo cells than in LoVo/Dx cells (Figure 3). All phenothiazine derivatives, apart from APh-FLU, increased Dox accumulation when applied at the concentration of 2.5 μM. At the same concentration, simvastatin raised the accumulation of the anticancer drug to a similar extent. Further increase of Dox intracellular accumulation was observed when phenothiazine derivatives were combined with simvastatin. The level of drug accumulation recorded in the case of all two-component mixtures was significantly higher than the one noted in the presence of phenothiazine derivative alone.

### 2.3. ABCB1 Expression

**Figure 4:**
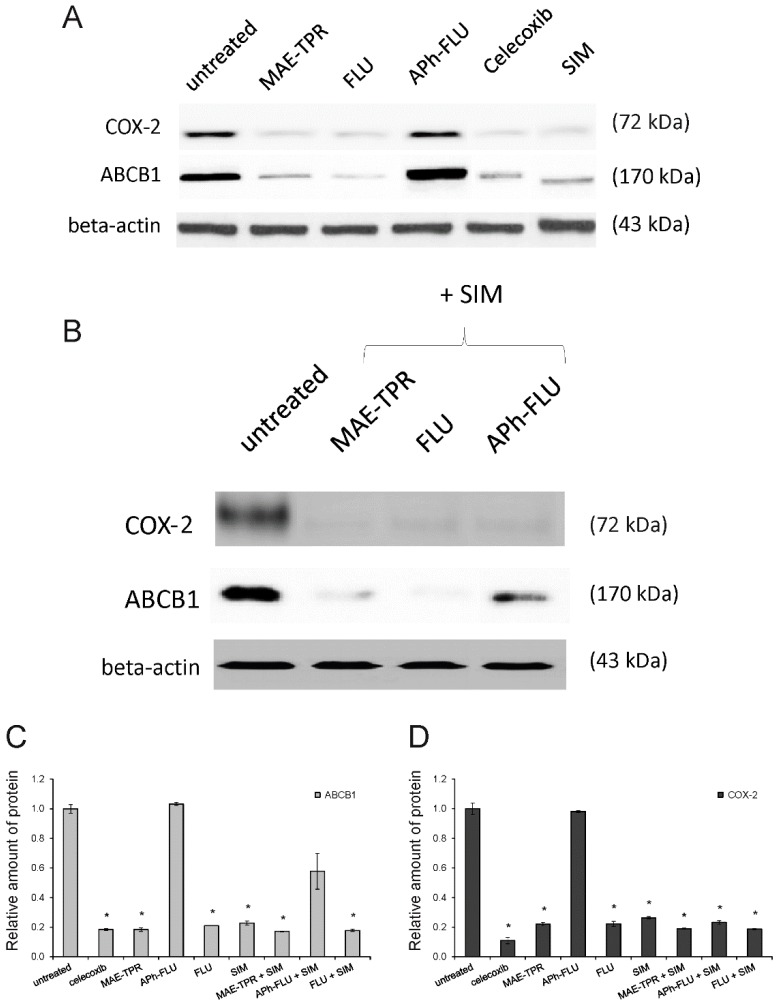
Analysis of cyclooxygenase-2 (COX-2) (dark grey bars) and ABCB1 proteins (light grey bars) expression in LoVo/Dx cells treated with phenothiazine derivatives and simvastatin (SIM) as single agents (**A**) and phenothiazine derivatives in combination with simvastatin (**B**) for 48 h. The molecular masses of the proteins are indicated at the right side of the gel. β-actin was used as a reference protein. Celecoxib (selective COX-2 inhibitor) was used as a control. The relative level of ABCB1 (**C**) and COX-2 expression (**D**) normalized to the control derived from non-treated LoVo/Dx cells. The results of three experiments ± SD are presented. The statistically significant differences from the untreated controls were determined using Student’s *t*-test (* *p* < 0.05).

**Figure 5:**
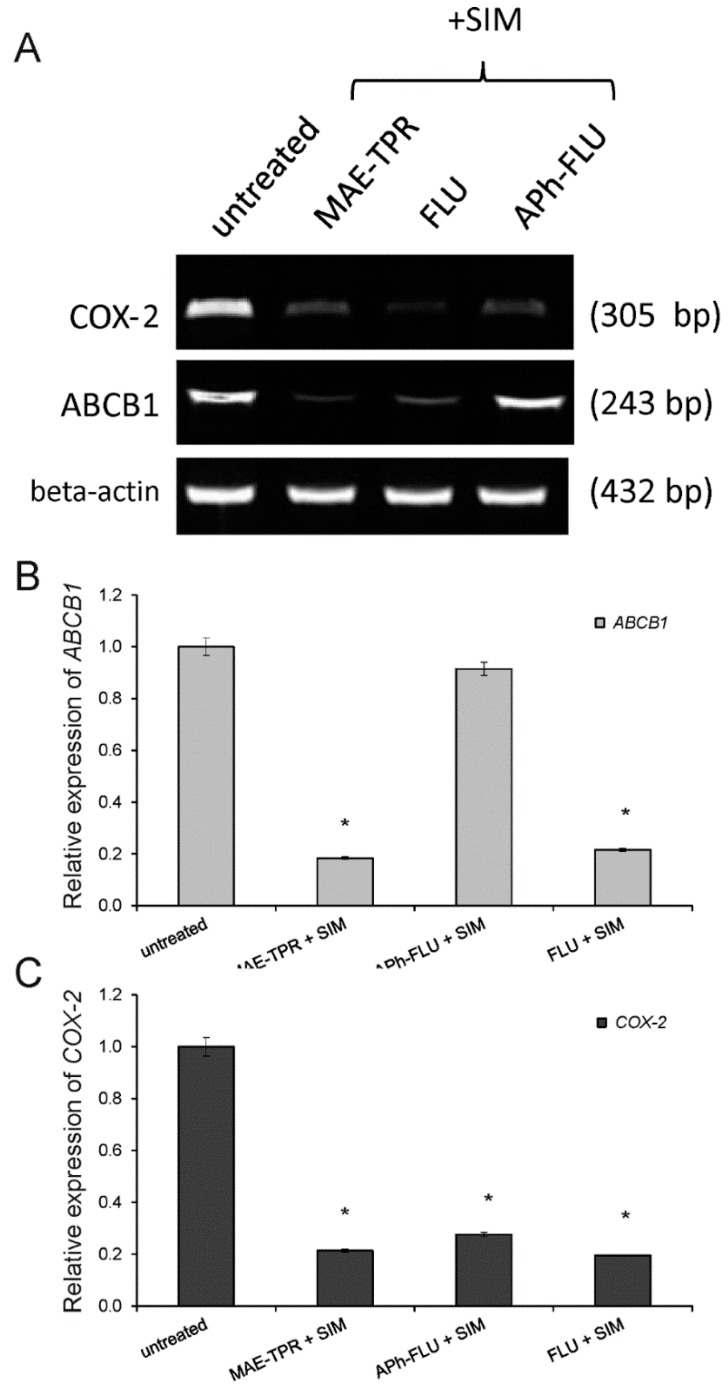
Analysis of *COX-2* (dark grey bars) and *ABCB1* genes (light grey bars) expression in LoVo/Dx cells cultured with phenothiazine derivatives and simvastatin (SIM) in combination (**A**) for 48 h. The base pair lengths of the amplified products are indicated at the right side of the gel. β-actin was used as a reference gene. The relative level of *ABCB1* (**B**) and *COX-2* expression (**C**) normalized to the control derived from non-treated LoVo/Dx cells. The results of three experiments ± SD are presented. The statistically significant differences from the untreated controls were determined using Student’s *t*-test (* *p* < 0.05).

It has been previously shown that drug sensitive LoVo cells differed from their Dox-resistant counterparts, LoVo/Dx cells, by the lower expression of ABCB1 transporter [23,25]. The incubation of LoVo/Dx cells in the presence of MAE-TPR, FLU, or simvastatin at the concentration of 2.5 μM resulted in a significantly decreased level of ABCB1 protein expression as compared to untreated cells (Figure 4A,C). Only APh-FLU exerted no effect on ABCB1 expression. When phenothiazine derivatives were combined with simvastatin, the reduction of expression of ABCB1 transporter was observed for all of the studied compounds again, except for APh-FLU (Figure 4B,C). The same results for combinations of phenothiazine derivatives with simvastatin were also observed at the mRNA level (Figure 5A,B).

### 2.4. COX-2 Expression

The difference in the expression of COX-2 between LoVo and LoVo/Dx cells has also been observed in our previous studies [26]. A higher level of the inducible form of the enzyme characterized the Dox-resistant cells. Here, the influence of phenothiazine derivatives and simvastatin (all compounds used at 2.5 μM concentration) on the expression of this protein in LoVo/Dx cells was investigated (Figure 4A,D). Celecoxib, a specific inhibitor of COX2, was used as a positive control. MAE-TPR, FLU, and simvastatin significantly and similarly reduced the level of COX-2, whereas APh-FLU had no effect on the expression of this enzyme. Co-treatment of LoVo/Dx cells with phenothiazine derivatives together with simvastatin resulted in diminished expression of COX-2 in all cases both at the protein (Figure 4B,D) and mRNA level (Figure 5A,C).

### 2.5. COX-2 Activity

**Figure 6:**
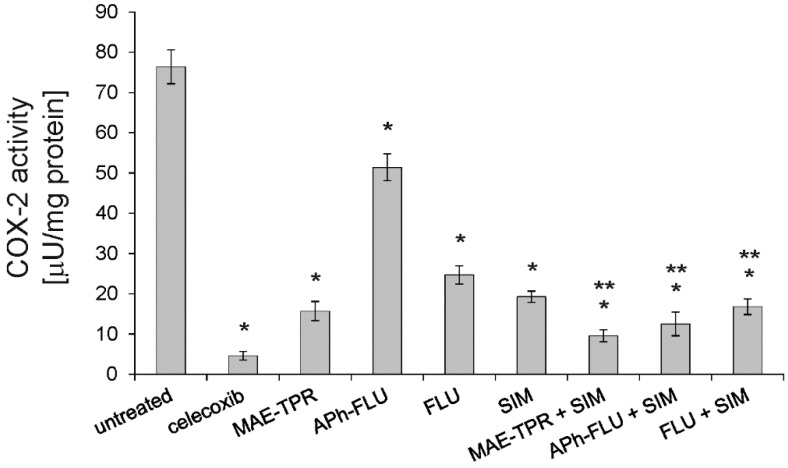
COX-2 activity in LoVo/Dx cells treated with phenothiazine derivatives, simvastatin (SIM) and phenothiazine derivatives in combination with simvastatin. Means of three experiments ± SD are presented. The statistically significant differences from the untreated LoVo/Dx control were determined using Student’s *t*-test (* *p* < 0.05). The statistically significant differences between the samples containing phenothiazine derivative as a single agent and samples with phenothiazine derivative combined with simvastatin were also determined using Student’s *t*-test (** *p* < 0.05).

When the influence of the studied phenothiazine derivatives on enzymatic activity of COX-2 was investigated, it turned out that all of them significantly reduced the activity of this enzyme when being applied at the concentration of 2.5 μM (Figure 6). MAE-TPR exerted the strongest effect, while the effect of APh-FLU was the weakest. Simvastatin that was applied as a single agent (at 2.5 μM) also diminished COX-2 activity. The combined application of phenothiazine derivatives, together with simvastatin, resulted in further decrease of COX-2 activity. This activity was significantly lower than in cells that were treated with phenothiazine derivatives alone.

### 2.6. Bcl-2 and Bax Expression

**Figure 7:**
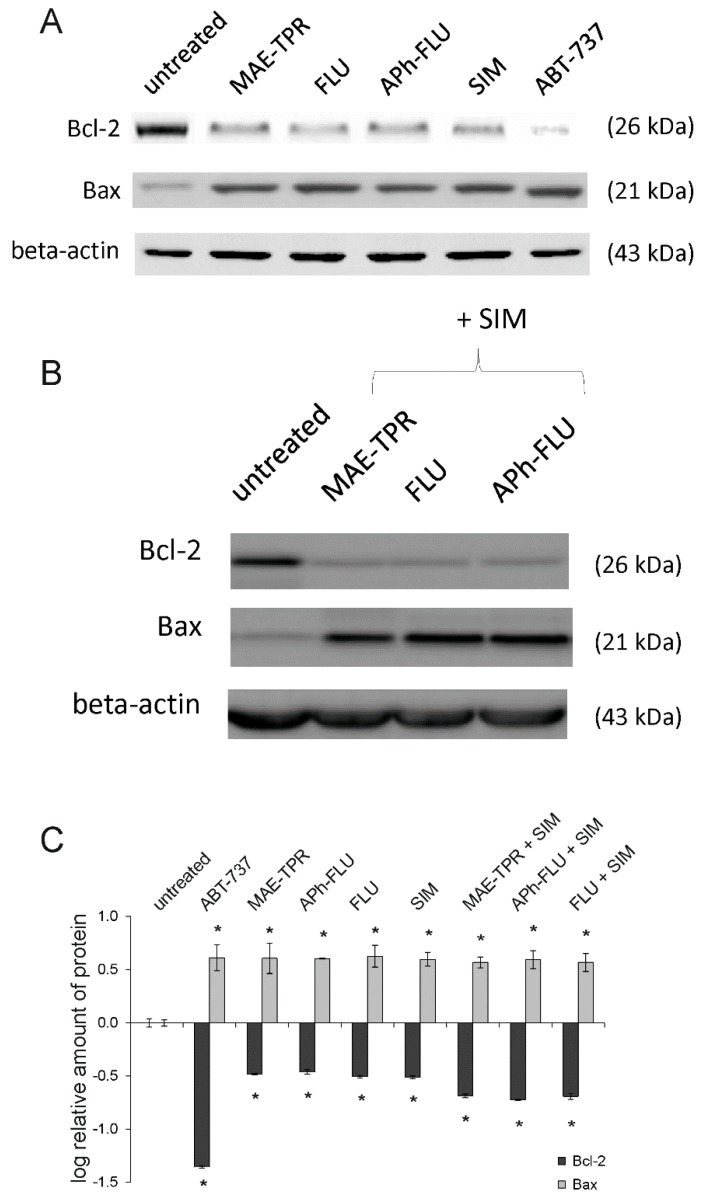
Analysis of Bcl-2 (dark grey bars) and Bax proteins (light grey bars) expression in LoVo/Dx cells cultured for 48 hours with phenothiazine derivatives and simvastatin (SIM) as single agents (**A**) and phenothiazine derivatives in combination with simvastatin (**B**). The molecular masses of the proteins are indicated at the right side of the gel. β-actin was used as a reference protein. ABT-737 was used as a positive control. The log of relative level of Bcl-2 and Bax expression (**C**) normalized to the control derived from non-treated LoVo/Dx cells. The results of three experiments ± SD are presented. The statistically significant differences from the untreated controls were determined using the Student’s *t*-test (* *p* < 0.05).

It has been shown previously that MAE-TPR, APh-FLU, and FLU induced apoptosis in LoVo and LoVo/Dx cells [24]. Similarly, pro-apoptotic properties of simvastatin in these cell lines were demonstrated [22]. Here, we have investigated whether phenothiazine derivatives alone or in combination with simvastatin influenced the level of expression of Bax and Bcl-2, the proteins engaged in regulation of apoptosis. As shown in Figure 7A,C, all of the phenothiazine derivatives and simvastatin (applied at 2.5 μM concentration) reduced the expression of anti-apoptotic Bcl-2 protein, and at the same time, increased the expression of pro-apoptotic Bax. The combination of phenothiazine derivatives, together with simvastatin, did not further increase Bax expression (Figure 7B,C). LoVo/Dx cells that were treated with phenothiazine:simvastatin mixture were characterized by slightly lower level of Bcl-2 expression than the cells treated by single modulators, however the effect was found to be statistically insignificant (Figure 7B,C). ABT-737, which is a Bcl-2 inhibitor, was used as a positive control.

### 2.7. Caspase-3 Activity

**Figure 8:**
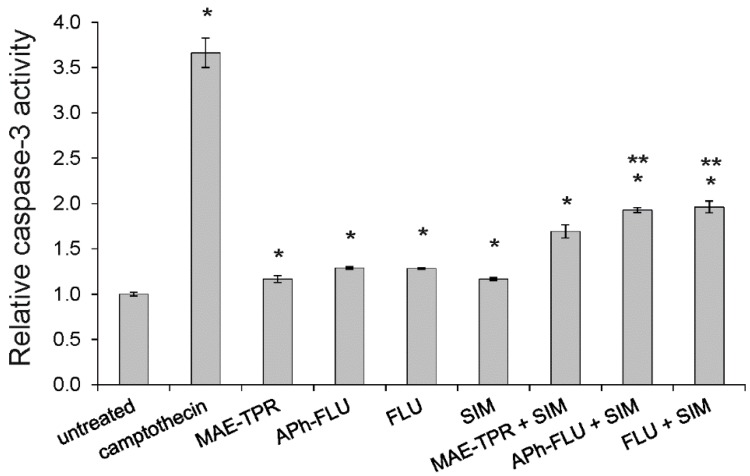
Relative caspase-3 activity in LoVo/Dx cells that were treated with phenothiazine derivatives, simvastatin (SIM), and phenothiazine derivatives in combination with simvastatin. Means of three experiments ± SD are presented. The statistically significant differences from the untreated LoVo/Dx control were determined using Student’s *t*-test (* *p* < 0.05). The statistically significant differences between samples containing phenothiazine derivative as a single agent and samples with phenothiazine derivative combined with simvastatin were also determined using the Student’s *t*-test (** *p* < 0.05).

Additionally, the influence of the studied modulators on the activity of caspase-3, which is a key enzyme of apoptosis execution phase, was investigated. When LoVo/Dx cells were treated by either phenothiazine derivatives or simvastatin (all modulators at 2.5 μM), an increase of caspase-3 activity was recorded (Figure 8). Further increase of enzymatic activity was recorded when co-treatment by phenothiazine derivatives with simvastatin was applied. In case of APh-FLU and FLU, but not MAE-TPR, the addition of simvastatin resulted in the increase of caspase-3 activity to a level that is significantly higher than in case in which the phenothiazine derivative was applied alone.

## 3. Discussion

The study on the effect of phenothiazine derivatives on the cytotoxicity of Dox on LoVo/Dx cells that are partially resistant to this anticancer drug revealed that MAE-TPR significantly increased Dox cytotoxic potential, the activity of FLU was minimal, while APh-FLU was inactive in this respect. At the same time, MAE-TPR and FLU, but not APh-FLU, were able to increase intracellular Dox accumulation as well as reduce the level of expression of ABCB1 transporter protein. These results pointed to MAE-TPR in being an effective Dox-resistance reversing agent in LoVo/Dx cells. This is consistent with our previous observations [23]. FLU was previously shown to be a weak modulator of resistance to Dox, vinblastine, and vincristine in a panel of drug resistant cancer cell lines [27]. Additionally, other phenothiazine derivatives, either commercially available as trifluoperazine [14] and thioridazine [15] or newly synthesized [28,29], proved to be effective MDR modulators in the in vitro models.

Simvastatin that was applied to LoVo/Dx cells as a single agent also caused an increase of Dox cytotoxicity and its intracellular accumulation. In our previous work, simvastatin-induced alteration in Dox accumulation was demonstrated by means of fluorescence microscopy [22]. The ability of statins to inhibit ABCB1 transporter has been previously observed in various model systems [30] and in drug-resistant cancer cell lines [31,32]. In the present work, it was also observed that simvastatin decreased the level of ABCB1 protein expression. Surprisingly, the increase in *ABCB1* (*Mdr1*) gene expression was previously observed to be exerted by simvastatin in LoVo/Dx cells [22]. The reason for this apparent discrepancy is likely to arise from very different simvastatin concentrations that were used in the two studies (2.5 μM here vs. 100 μM previously). On the other hand, there are contradictory reports regarding the influence of statins on the ABCB1 transporter expression. Atorvastatin was reported to reduce the expression of both mRNA and the protein of the ABCB1 transporter [33]; lovastatin did not affect the expression of *ABCB1* [32], whereas simvastatin was observed to induce the expression of this gene [34].

The most important result of the present study was the observation that the co-treatment of LoVo/Dx cells with phenothiazine derivatives and simvastatin resulted in the increase of Dox cytotoxicity to these cells. In all cases, Dox was significantly more cytotoxic to cancer cells that were treated by a two-component drug mixture as compared to cells treated by phenothiazine derivatives or simvastatin used as single agents. According to our best knowledge, this is the first demonstration of combined use of phenothiazine derivatives, together with a statin in cancer cells. Interestingly, methylene blue, a drug with a structure that resembles phenothiazine, was demonstrated to show synergy with atorvastatin in killing *Plasmodium falciparum* cells [35].

When the influence of phenothiazine derivatives and simvastatin on COX-2 activity and expression was investigated, it was noticed that all studied compounds reduced the activity of this enzyme. Simvastatin and all phenothiazine derivatives, apart from APh-FLU, also reduced the expression of COX-2 protein. The combined treatment of LoVo/Dx cells with phenothiazine derivatives, together with simvastatin, resulted in further decrease of COX-2 activity. The reduction of COX-2 protein expression was, however, similar in the case of single agent-treatment and in the case of application of phenothiazine derivatives:simvastatin mixture. Some phenothiazine derivatives have been previously reported to possess anti-inflammatory activity, however the majority of reports came from animal studies and the molecular mechanism of their anti-inflammatory action was not discussed [36,37]. The approach, which was based both on computational and combinatorial chemistry methods, yielded a phenothiazine-type lead compound that was a selective COX-2 inhibitor [38]. Recently, a series of compounds bearing a phenothiazine-scaffold were synthesized and found to possess antioxidant, anti-inflammatory (via cyclooxygenase inhibition), and hypolipidemic (via squalene synthase inhibition) activities [39]. The ability of simvastatin to affect COX-2 expression and activity is better recognized. Among others, simvastatin was shown to diminish COX-2 expression in human monocytes [40], in both healthy and scrapie-infected mouse brain tissue [41], in the mouse model of acute colitis [42], as well as in a model of chronic kidney disease (human mesangial cells stimulated by angiotensin II) [43].

The studies on apoptosis induction revealed that phenothiazine derivatives and simvastatin, when used as single agents (at 2.5 μM concentration), activated caspase-3, the main apoptosis-executing protease. The combination of phenothiazine derivatives with simvastatin resulted in the further increase of caspase-3 activity that, in the case of APh-FLU and FLU, was significantly higher than when phenothiazine derivatives were applied alone. All the studied modulators affected the expression of two important apoptosis regulators, Bcl-2 and Bax proteins. The level of anti-apoptotic Bcl-2 was diminished, whereas pro-apoptotic Bax was upregulated. The co-treatment of LoVo/Dx cells with phenothiazine derivatives and simvastatin did not further influence the expression level of both Bcl-2 and Bax. Apoptosis induction in LoVo/Dx cells by the studied phenothiazine derivatives has been previously demonstrated by DNA fragmentation, disruption of actin filament organization, poly(ADP ribose) polymerase (PARP) cleavage and caspase-3 activation [24]. The ability of phenothiazine derivatives to induce apoptosis in cancer cells has been thoroughly characterized (reviewed in [44]). Such derivatives as perphenazine, FLU, thioridazine, and trifluoperazine were observed to activate caspase-3 in various cancer cells [45,46,47]. Thioridazine and trifluoperazine were also shown to promote cell apoptosis via the down-regulation of the expression of Bcl-2 and the up-regulation of the expression of Bax [48,49]. Additionally, the pro-apoptotic activity of statins has been often reported. Simvastatin was demonstrated to activate many caspases, including caspase-3 [20,50,51]. The reduced expression of Bcl-2 accompanied by the increased expression of Bax was observed in the presence of simvastatin in lymphoma cells [50] as well as in the colon [20] and breast cancer cells [51,52].

According to the initial assumption that the combined use of two MDR modulators of dissimilar mechanism of action would be beneficial, it was demonstrated that the combined application of phenothiazine derivatives and simvastatin caused a strong reduction of Dox resistance in human colon adenocarcinoma cells. Apart from influencing anti-MDR potency of phenothiazine derivatives, simvastatin also strengthened their anti-inflammatory and pro-apoptotic properties. Statins have been already observed to enhance the effectiveness of different types of anticancer therapies. Statins were able to potentiate anti-tumor effects of various chemotherapeutics (such as anthracyclines, platinum compounds, 5-fluorouracil, etoposide, paclitaxel, and cytosine arabinoside), immunotherapy, as well as radiotherapy [53,54]. Additionally, the combination of statins with cyclooxygenase inhibitors potentiated their anti-proliferative and pro-apoptotic activity [26,55,56], and subsequently reduced cancer progression in a model system of chemically-induced carcinogenesis [57]. Since statins demonstrate pleiotropic biological activity, multiple molecular mechanisms have been associated with the therapy-enhancing effects of statins. Therefore, further studies are required to find an accurate explanation of the observed potentiation of Dox-resistance reversal properties of phenothiazine derivatives by simvastatin.

## 4. Materials and methods

### 4.1. Chemicals

MAE-TPR: 10-[3-(*N*-2-hydroxyethyl-*N*-methylamino)-2-hydroxypropyl]-2-trifluoromethylphenothiazine and APh-FLU 10-{3-[4-(4-acetylphenyl) piperazin-1-yl]-2-hydroxypropyl}-2-trifluoromethylphenothiazine dihydrochloride were synthesized, as described previously [24,58]. Fluphenazine (FLU), simvastatin, sulforhodamine B, ABT-737, camptothecin, and Dox were purchased from Sigma-Aldrich (Poznan, Poland). ABT-737, phenothiazine derivatives and simvastatin used in the experiments were dissolved in DMSO. Dox was dissolved in water. Unless indicated otherwise, all phenothiazine derivatives and simvastatin were used in a concentration of 2.5 μM.

### 4.2. Cell Lines

The human colorectal adenocarcinoma cell line LoVo and its doxorubicin-resistant subline LoVo/Dx [59,60] were obtained from Institute of Immunology and Experimental Therapy of Polish Academy of Science (Wroclaw, Poland). The identity of the cell lines used was confirmed by STR analysis (STRA7227, January 2018). The cells were incubated in a humidified atmosphere (5% CO_2_, 95% air) at 37 °C and then cultured in F12 medium (Cytogen, Wetzlar, Germany) supplemented with 10 % fetal bovine serum (Gibco, Waltham, MA, USA), 1% antibiotic antimycotic solution (Sigma-Aldrich, Poznan, Poland), and 1% glutamine (Sigma-Aldrich). Dox (100 ng/mL) was added to the medium to maintain drug resistance of LoVo/Dx cells after each passage, which was carried out twice a week. All of the procedures were carried out in log-phase of cell growth. The adherent cells were removed from the flask surface using non-enzymatic cell dissociation solution (Sigma-Aldrich). The density of the cells for each experiment was determined with EVE Automatic Cell Counter (NanoEnTek, Seoul, Korea).

### 4.3. Cell Viability Assay

The sulforhodamine B (SRB) assay was performed, as previously described [61], with modifications. Shortly, 30,000 of LoVo/Dx cells were seeded in 96 well plates and given a 60 min attachment period (at 37 °C). Thereafter, the cells were treated with the studied compounds in the appropriate concentrations for 48 h. Control wells only contained medium. The further procedure was carried out as previously described [62]. Cytotoxicity of DMSO to LoVo and LoVo/Dx cells was found to be negligible.

### 4.4. Isobolographic Analysis

By the CompuSyn software according to the classic median-effect equation, as described by Chou and Martin, the combination index (CI) values were calculated [63].

(1) $$\mathrm{CI}=\frac{{\left(D\right)}_{1}}{{\left(\mathrm{Dx}\right)}_{1}}+\frac{{\left(D\right)}_{2}}{{\left(\mathrm{Dx}\right)}_{2}}$$

where: (Dx)_1_ is the dose of drug 1 alone that inhibits a system by x%, (Dx)_2_ is the dose of drug 2 alone that inhibits a system by x%, and (D)_1_ + (D)_2_ are the doses of drug 1 and 2 in combination that also inhibit a system by x%. CI values below 1 represent synergism, CI values equal to 1 indicate additive effect (i.e., no interaction), and CI values above 1 point to antagonism.

### 4.5. Intracellular Accumulation of Doxorubicin

Intracellular Dox accumulation was detected with a fluorimetry based assay, as described previously [64], with minor modifications. In brief, the cells were seeded (800,000/well) onto a six-well plate in 2 mL of fresh medium without Dox and incubated for 24 h at 37 °C. Subsequently, the cells were incubated in PBS containing Dox at the concentration of 4 μM and treated with the modulators alone or in combination with simvastatin. After 48 h of incubation, the cells were washed twice in ice-cold PBS and detached with trypsin/EDTA (0.05/0.02% *v*/*v*). Next, cells were centrifuged (30 s, 13,000× *g*, 4 °C) and resuspended in 1 mL of a 1:1 mixture of ethanol/0.3 N HCl. 50 μL of cell suspension were sonicated on ice with two 10 s bursts (Labsonic sonicator, 100 W) and used for the measurement of cellular proteins. The protein content was determined using the standard method of Bradford reaction [65]. Dox content was measured using Perkin-Elmer LS-5 spectrofluorimeter (Perkin-Elmer, Beaconsfield, UK). Excitation and emission wavelengths were 475 and 553 nm, respectively. Fluorescence was expressed in ng of Dox per mg of cellular protein with the use of the calibration curve that was prepared previously.

### 4.6. Caspase 3 Activation Assay

Caspase-3 activation was estimated with the use of a commercially available kit (GenScript, Piscataway, NJ, USA). The cells were seeded (800,000/well) onto a six-well plate in 2 mL of medium and incubated for 24 h at 37 °C. Then, the cells were treated with the modulators alone, and in combination with simvastatin. After incubation with the studied compounds for 48 h, the cells were scraped and centrifuged (2000× *g*, 5 min, 25 °C). Spectrophotometric detection (A_405_) of the chromophore p-nitroanilide (pNA) was used to measure caspase-3 activity. The relative increase of caspase-3 activity was determined by calculating the ratio of the absorbance of pNA in the studied sample (treated with the modulator) to the control (with no modulator).

### 4.7. Western Blot Analysis

The expression of studied protein in LoVo/Dx was performed, as previously described [24]. Shortly, ice-cold lysis buffer (1% Triton X-100, 50 mM Hepes, 150 mM NaCl, 1.5 mM MgCl_2_, 1 mM EGTA, 1 mM phenylmethylsulfonyl fluoride (PMSF),100 mM NaF, 10 mM sodium pyrophosphate, 10 μg/mL aprotinin, and and 10% glycerol, pH 7.4) was used to cell lysis. After centrifugation (13,000× *g*, 10 min, 4 °C), the supernatants were collected for analysis. The standard method of Bradford reaction [65] was used to determine the protein content. The proteins were separated by SDS-PAGE, transferred onto polyvinylidene difluoride (PVDF) membranes, and detected using primary antibodies in TBS-T buffer (0.1% Triton X-100, 50 mM Tris-HCl, 150 mM NaCl, pH 7.4) containing 5% bovine serum albumin (BSA).

The following primary antibodies were used: mouse Anti-Cox-2 Antibody (dilution 1:1000, BD Biosciences, Franklin Lakes, NJ, USA), mouse Anti-Bcl-2 antibody (100/D5; dilution 1:500, (Abcam, Cambridge, UK), mouse anti-Bax antibody (2D2; dilution 1:600, Abcam), and anti-ABCB1 mouse monoclonal primary antibody (C494 dilution 1:1000, Alexis Biochemicals, San Diego, CA, USA). The level of β-actin was also determined (mouse monoclonal anti-actin antibodies (C4, dilution 1:5000, Santa Cruz Biotechnology, Dallas, TX, USA) as a reference protein. After incubation (overnight at 4 °C), the membranes were washed in TBS-T and then incubated with rabbit anti-mouse IgG secondary antibody that was conjugated to horse radish peroxidase (HRP) (dilution 1:1000, Thermo Scientific, Waltham, MA, USA) for 30 min at 4 °C. The membranes were then washed with TBS-T and the proteins were visualized. The relative level of protein normalized to the control derived from non-treated-cells was determined. The Image J program was used to detect the optical density of the bands on the electrophoregrams.

### 4.8. Polymerase Chain Reaction

Cells were seeded onto six-well plates and allowed to attach onto the plate surface (24 h, 37 °C). Next, the studied compounds were added and the cells were incubated for further 48 h at 37 °C. RNA extraction and reverse transcription was performed as described previously [23]. The primers for *ABCB1*, *COX-2* and *β-actin* (a reference gene) were designed as presented: *β-actin* primers: 5′-TGAGCGCGGCTACAGCTT-3′ and 5′-TCCTTAAT-GTCACGCACGATTT-3′; *ABCB1* primers: 5′-AAGCTTAGTACCAAAGAGGCTCTG-3′ and 5′-GGCTAGAAACAATAGTGAAAACAA-3′; *COX-2* primers: 5′-CCGGGTACAATCGCACTTAT-3′ and 5′-GGCGCTCAGCCATACAG-3′. All of the sequences were synthesized in the Institute of Biochemistry and Biophysics of Polish Academy of Science (Warsaw, Poland). The amplification was carried out, as described previously [23]. To visualize the separated fragments, the Gel Documented System KODAK MI v 4.0.0 was used. The relative level of *ABCB1* and *COX-2* expression normalized to the control was estimated by the detection of optical density of the bands on the electrophoregrams (Image J program).

### 4.9. Cyclooxygenase (COX-2) Assay

COX-2 assay was performed following the recommendations of the manufacturer (Fluorometric Cyclooxygenase Activity Assay Kit, Abcam). In brief, the cells were seeded (800,000/well) onto a six-well plate in 2 ml of medium and incubated for 24 h at 37 °C. Subsequently, the cells were treated with the modulators alone, and in combination with simvastatin. The studied compounds were added in a fresh portion of medium and the incubation was continued for 48 h. Cells were harvested, counted (3 × 10^6^ cells for each sample) and washed with phosphate-buffered saline (PBS). After centrifugation (500× *g*, 3 min), the pellets were resuspended in 0.5 mL of lysis buffer with protease inhibitor cocktail. The cell lysate was centrifuged (12,000× *g*, 4 °C, 3 min) and supernatant was collected. To initiate the reaction, the arachidonic acid/NaOH solution was added into each sample. After a series of reaction mediated by cyclooxygenase, a highly fluorescent compound, resorufin, was generated in the samples. Celecoxib, a specific inhibitor of COX-2, was used as a positive control. After the reaction, fluorescence intensity in samples was recorded (Ex/Em = 535/587 nm). Activity of COX-2 in the test samples was calculated as:

(2) $$\mathrm{COX}\;\mathrm{activity}=\frac{B}{\Delta T\times M}$$

where:

B—amount of resorufin from standard curve;
ΔT—reaction time;
M—protein amount added into the reaction well.

### 4.10. Data Analysis

All of the experiments were repeated three times. Data represent the mean ± standard deviation (SD) of at least three replications. Student’s *t*-test was applied and *p*-values less than 0.05 were considered to achieve statistical significance.

## Figures and Tables

**Table 1:** Combination of phenothiazine derivatives and simvastatin (SIM) with doxorubicin (Dox) against LoVo/Dx cell growth.

Concentration [M]			Ratio	Combination Index
Dox	MAE-TPR	SIM		(CI)
10	2.5		4:1	0.2261
10		2.5	4:1	0.4092
10	2.5	2.5	4:1:1	0.0748
**Dox**	**APh-Flu**	**SIM**		
10	2.5		4:1	1.0101
10	2.5	2.5	4:1:1	0.2116
**Dox**	**FLU**	**SIM**		
10	2.5		4:1	0.8261
10	2.5	2.5	4:1:1	0.348

Dose and effect data were obtained from the sulforhodamine B (SRB) assay (mean values of three experiments) and analyzed by CompuSyn software. CI values were calculated by CompuSyn software. CI = 1 indicates additive effect, CI < 1—synergism, and CI > 1—antagonism.
